# Early Intervention Service Delivery via Telehealth During COVID-19: A Research-Practice Partnership

**DOI:** 10.5195/ijt.2021.6363

**Published:** 2021-06-22

**Authors:** Jessica Kronberg, Elaine Tierney, Anna Wallisch, Lauren M. Little

**Affiliations:** 1 M Street Pediatric Therapy, Chicago, Illinois, USA; 2 Juniper Gardens Children’s Project, University of Kansas, Kansas City, Kansas, USA; 3 Department of Occupational Therapy, Rush University Medical Center, Chicago, Illinois, USA

**Keywords:** Community-based, Early Intervention, Family-focused, Occupational Therapy, Research-Practice Partnerships, Telehealth

## Abstract

Coaching has been identified as a best practice for early intervention (EI) services provided through the Individuals with Disabilities Education Act (IDEA) Part C. The current study describes the establishment and progress of a research-relationship partnership to deliver coaching via telehealth during the COVID-19 pandemic. Community-based EI providers implemented 9-weeks of telehealth coaching and evaluated the extent to which child and caregiver outcomes differed between families that had previously received in-person services versus telehealth only. Four EI providers completed the intervention with n=17 families of children aged 6-34 months during the pandemic (April-August 2020). We used the Canadian Occupational Performance Measure (COPM) and Goal Attainment Scaling (GAS) to collect outcomes on caregiver identified goals; we used Wilcoxon Signed Rank Tests to examine pre- to post-intervention data. Results showed significant improvements in parent satisfaction, child performance, and goal attainment (all *p*<.01). Findings suggest that telehealth coaching procedures implemented by community-based EI providers resulted in improvements in caregiver identified goals for young children.

Due to the COVID-19 pandemic, telehealth rapidly expanded as a service delivery model across state early intervention (EI) systems. Telehealth is the use of information and communication technology (ICT) to support access to care (American Academy of Pediatrics, 2020). While ICT may encompass both synchronous and asynchronous methods to deliver care, we refer to the use of synchronous videoconferencing methods as ‘telehealth’ within this article. Extant evidence shows that EI positively impacts children's developmental trajectories (e.g., Klintwall et al., 2015) and family resilience (Twoy et al., 2007). During the pandemic, it was vital to maintain families’ access to EI services through a telehealth service delivery model. While some statewide EI systems have implemented telehealth in EI prior to the pandemic (e.g., Cole et al., 2016; Cole et al., 2019), the outcomes of the sudden adoption of telehealth use for all EI providers and families during the pandemic remains unknown.

Early childhood coaching (Rush & Sheldon, 2011) has been identified as a best practice method of EI service delivery (Adams & Tapia, 2013). Early childhood coaching draws on best practice principles related to family centered care and is rooted in increasing caregiver and family capacity (Division for Early Childhood [DEC], 2014). Research suggests that barriers to the implementation and evaluation of coaching in “real-world” EI practice persist. For example, Douglas et al., (2020) reported that EI providers had experienced difficulty in explaining the process of coaching and engaging parents. Additionally, some aspects of coaching (i.e., modeling, joint planning) were viewed as most important to EI providers.

Research shows that telehealth may be an efficacious model to serve families in EI (Cason, 2009; 2011), including families of young children with various neurodevelopmental conditions including autism spectrum disorders (Sutherland et al., 2018) and Fragile X Syndrome (e.g., Hall et al., 2020). Coaching delivered through telehealth has been shown to significantly improve child participation and parent self-efficacy (Little et al., 2018), and be highly acceptable to families (Wallisch et al., 2019). However, data on the efficacy of coaching delivered via telehealth for families of young children with neurodevelopmental conditions has largely been based in research studies with strict interventionist training and participants who are interested in research (e.g., Ciupe & Salisbury, 2020), potentially reducing the generalizability of findings. The perceived barriers to early childhood coaching described by community-based interventionists may be magnified in the telehealth model; the coaching components of reflection and observation may take the bulk of intervention time in a telehealth session and modeling is either limited or absent. Additionally, the efficacy of coaching via telehealth in real world settings for families that previously received in-person services remains unknown.

The COVID-19 pandemic presented the opportunity to research the efficacy of a rapid expansion of telehealth practice from a community-based perspective. To date, the number of effective interventions for children with disabilities far outweighs the number of interventions actually used in practice, and it often takes an average of 17 years before evidence-based practices are used in practice (Morris et al., 2011). To reduce this gap, it is critical to develop research-practice partnerships (RPPs) in the community to more rapidly increase the uptake of interventions. RPPs involve long-term, trusting, and collaborative relationships that equally value the opinions of both practitioners and researchers (Tseng et al., 2017). RPPs occur when practitioners and researchers co-design studies to implement a novel intervention within a community organization and are linked to better outcomes for children. Strong RPPs are an initial step towards changing standard practices and ultimately ensuring children in EI settings are receiving evidence-based interventions. While telehealth was used as a model for EI services in some states prior to the pandemic, few studies have examined how coaching via telehealth may be translated into practice using RPPs.

Our team, comprised of two researchers and two community-based EI providers, engaged in collaborative efforts to increase the use of early childhood coaching in telehealth. Prior to the COVID-19 pandemic, an EI organization, part of a midwestern state-wide EI network, became interested in expanding their telehealth practice. A collaborative partnership was developed to train an EI occupational therapy practitioner (first author) on the coaching via telehealth model described above. As part of the training, this EI provider was trained to use telehealth to provide EI services for a research study led by the last author, who provided ongoing feedback, including fidelity checks. This EI provider began training other providers in the EI organization after completing the research study. When the COVID-19 pandemic occurred, all EI providers in the state completed coaching via telehealth training provided by the third and fourth authors and received a certificate of completion prior to providing EI services through telehealth in April 2020. At the same time, additional EI providers from this partnering community organization were trained in Goal Attainment Scaling (GAS; Kiresuk & Sherman, 1968) and the Canadian Occupational Performance Measure (COPM; Law et al., 1994). The EI providers that participated in this 90-minute training were interested in using the GAS and COPM for the purpose of gathering outcome data from families enrolled in EI provided via telehealth.

The current study extends previous studies of coaching and telehealth in numerous ways. First, previous studies used strictly trained interventionists whereas the current intervention was delivered by community-based EI providers that attended a 2.5 hour coaching training and a 1.5 hour outcome measure training, and received one-on-one support from the trained fidelity occupational therapy practitioner. Second, previous studies of coaching have used primarily homogenous samples based on diagnosis (e.g., Little et al., 2018); the current study used a range of children enrolled in EI services with various diagnoses. Third, many families enrolled in telehealth research interventions have previously received in-person therapies, limiting the ability to examine the extent to which families that have never received in-person services achieve goals as identified in a coaching intervention. Therefore, the purpose of this study was to investigate the efficacy of a 9-week coaching telehealth intervention for families engaged in a community-based, statewide EI system among a small sample of EI providers and families. Specific research questions included: (1) To what extent do caregivers of children in EI report satisfaction and child performance in individualized goals over a 9-week telehealth coaching intervention? and (2) To what extent does previous experience with EI (in-home vs. telehealth only) influence parent reported outcomes?

## METHOD

The current study was initiated by a community-based EI agency at the onset of telehealth use during the pandemic. Using a permission form developed by the community agency, EI providers asked families if they would like to participate in a data tracking project on parent and child outcomes resulting from participation in telehealth delivered EI services during the pandemic. The community agency then partnered with the research team for training in outcome measures. The research team then used a retrospective data review of families enrolled in telehealth EI services at the onset of the pandemic (i.e., March-June 2020). The data used for analyses were de-identified and the study was approved by a University Institutional Review Board as a quality improvement project.

## INTERVENTION

After participating in training opportunities, four EI providers delivered the coaching intervention, including one physical therapist, two occupational therapists, and one speech-language pathologist. Coaching trainings were rooted in Early Childhood Coaching (Rush & Sheldon, 2011), Division for Early Childhood Recommended Practices (2014), and Occupation-Based Coaching (Dunn et al., 2018). When caregivers have opportunities to problem solve through the situation in which they encounter with their children every day, they then have the capacity to troubleshoot activities to increase children's participation and subsequent learning opportunities (Rush & Sheldon, 2011). The early child coaching model demands that the parent serves as the expert and the EI provider serves as the coach, only offering information and strategies with permission and supporting the caregiver in their own identified goals and problem-solving process. The early childhood coaching process consists of (1) Joint Planning, (2) Observation, (3) Action (in which modeling often occurs), (4) Reflection, and (5) Feedback. Caregivers are invited to draw on children's strengths to maximize children's learning opportunities and reframe challenges. During telehealth sessions, EI providers must often shift their use of modeling to increasingly focus on engaging caregivers in observation and reflection.

## PARTICIPANTS

Families (n=17) were enrolled in the state EI program; children ranged from ages 6-34 months (M=20.18 months, SD=10.33 months). Seven (42%) of families were Hispanic/Latino, nine (52.9%) families were White, non-Hispanic and one (5.9%) family preferred to not report race/ethnicity. Eight families received services through Medicaid (47.1%); primary languages included English (12, 70.6%), Spanish (3, 17.6%), and other languages (2, 11.8%). Families that spoke Spanish and other languages used a translator for EI service delivery. Child diagnoses included developmental delay, genetic disorders, and expressive/receptive language delay. Seven families were enrolled in EI during the pandemic and thus only experienced EI services via telehealth, whereas 10 families had received in-person EI services prior to transitioning to telehealth services.

## PROCEDURES

EI providers invited families to create specific, measurable short-term goals based on Individualized Family Service Plan (IFSP) outcomes using GAS (Kiresuk & Sherman, 1968) and the COPM (Law et al., 1994) (see Measures). Caregivers were asked to create 1-3 short-term goals during the first session and EI providers then used coaching to address the caregiver-identified goals throughout subsequent sessions. EI providers administered assessments at baseline and post-9 weeks of intervention, although families continued to receive EI services as part of their IFSP following the 9-week intervention period. Sessions ranged from 20–60 minutes. HIPAA-compliant (Health Insurance Portability and Accountability Act [HIPAA] of 1996, Pub. L. No. 104–191) videoconferencing platforms were used to deliver EI sessions through telehealth.

## MEASURES

Demographic measures were collected at baseline, and all interventionists had access to families’ IFSP reports. The below assessments were administered pre- and 9 weeks post-intervention.

*Goal Attainment Scaling* (GAS) is a method to track progress toward goals created by the individual, or in this case the family. Goals are placed on a 4-point scale with a scale from −2 (less than expected) to +1 (much more than expected). When a family identifies a goal for their child, the interventionist asks the family to describe specific child behaviors that would fall on a 4-point scale (−1: What does the child behavior look like now?; −2: What would the child behavior look like if it got worse?; 0: What would the behavior look like if it got slightly better?; +1 What would the behavior look like if it were perfect?). GAS demonstrates sound psychometric properties in pediatric focused studies (see Steenbeek, et al., 2007).

The *Canadian Occupational Performance Measure* (COPM; Law et al., 1998), a criterion referenced measure, assesses client-centered outcomes over time (Dedding et al., 2004). The COPM invites clients to rate their performance and satisfaction for each identified goal on a 10-point scale. Performance ranges from 1 (not able to perform) to 10 (performs extremely well), and satisfaction ranges from 1 (not at all satisfied) to 10 (extremely satisfied). Clinically meaningful change for COPM is a change in 2 points (Law et al., 1994). Combined use of the COPM and GAS has been shown to result in clinically significant improvements in self-identified goals (Doig et al., 2010; Trombly et al., 2002). The COPM was designed for use by occupational therapists. According to the authors, it can be used by multidisciplinary teams as an intake tool or to evaluate other domains of concern. However, this shift in use results in it no longer being the original COPM (COPM, n.d.).

## DATA ANALYSIS

Descriptive analyses were used to examine commonalities among family-specified goals. The Wilcoxon Signed Rank Tests were used to examine differences in baseline to post-intervention scores in COPM Performance and COPM Satisfaction as well as GAS scores. We examined the combined COPM and GAS scores for each goal (i.e., 1–3 goals) and the collapsed scores across all goals. The Mann Whitney U tests were used to examine differences between those that had previously received in-person EI services (n=10) versus those that had only experienced telehealth (n=7) across the change scores of COPM child performance, COPM parent satisfaction, and GAS ratings.

## RESULTS

Descriptive analyses showed that goals included a variety of family centered goals across disciplines (see Table 1).

**Table 1:** Example Goals by Discipline

Discipline	Family Identified Goals*
Occupational Therapy	Eliminate breastfeeding for soothing
	Follow directions
	Maintain grasp on toys
	Reach for toys
	Potty training
	Use utensils
	Tolerate brushing teeth
	Assist in dressing
	Safety during routines
	Improve sleep participation
	Increase tolerance to textures
Physical Therapy	Move into sitting position
	Sit independently
	Walk down stairs
	Run
	Jump with both feet
	Crawl independently
	Stand independently
	Initiate rolling
	Reach for toys
Speech Therapy	Drink without spilling
	Use utensils
	Take smaller bites/limit over-stuffing
	Increase chewing skills
	Increase spontaneous use of words
	Use 2–3 words phrases

*Note.* *Goals overlapped among families

With regard to child participation outcomes (see Table 2), participants reported an increase in COPM child performance scores (*p*<.01) and COPM parent satisfaction scores (*p*<.01). Across all goals on the COPM, parents reported a mean increase in child performance of 2.97 (SD=1.99; range=−.33–7.50) and a mean increase in parent satisfaction of 2.42 (SD=2.77; range=−4.50–7.00). There was also a significant increase in GAS scores overall (*p*<.001). Refer to Table 2 for results related to individual goals (i.e., goals 1–3).

**Table 2:** COPM and GAS Results

	Change Scores Mdn (SD, range)	Wilcoxon Signed Rank	*p*
COPM Child Performance			
Overall	2.3 (1.99, −.33–7.50)	Z =−3.468	*p*<.01
Goal 1	3.0 (2.58, −3.0–7.0)	Z = −3.137	*p*<.01
Goal 2	3.0 (2.29, 0–8.0)	Z = −3.192	*p*<.01
Goal 3	2.0 (2.19, 0–6.0)	Z = −1.890	*p*=.059
COPM Parent Satisfaction			
Overall	3.0 (2.77, −4.50–7.0)	Z = −2.724	*p*<.01
Goal 1	3.0 (3.82, −9.0–8.0)	Z = −2.449	*p*<.05
Goal 2	3.0 (2.53, −3.0–7.0)	Z = −2.729	*p*<.01
Goal 3	2.0 (2.97, −2.0–6.0)	Z = −1.355	*p*=.176
Goal Attainment Scaling			
Overall	1.3 (.72 0–3)	Z = −3.528	*p*<.001
Goal 1	1.0 (.71, 0–3)	Z = −3.611	*p*<.001
Goal 2	1.0 (.83, 0–2.0)	Z = −2.879,	*p*<.01
Goal 3	0 (.89, 0–2.0)	Z = −1.342,	*p*=.180

*Note*. *COPM=Canadian Occupational Performance Measure

Mann Whitney U results showed a lack of significant differences between families that had previously experienced in-person EI services versus those that only experienced telehealth EI services for child performance Z=−1.03, p=.315, parent satisfaction Z=−1.075, p=.282, and GAS scores Z=−1.235, p=.230.

## DISCUSSION

Findings suggested that a 9-week coaching intervention delivered through telehealth by trained, community-based EI providers may be effective in promoting child goals and parent reported satisfaction with child gains. Short term coaching interventions have previously shown effectiveness in increasing child participation (Kessler & Graham, 2015; Ogourtsova et al., 2019). In a previous study using coaching over telehealth, families of children with ASD identified 2–3 goals over the course of 12 weeks (Little et al., 2018). Based on the current findings, it appears that similar results over 9 weeks can be achieved. Previous findings, however, point to at least two goals per family whereas some parents in the current study identified one goal to work on for the course of the 9 weeks. It may be that certain adaptive behavior domains may require more time and/or sessions for children to make gains. One meta-analysis indicated that adaptive behavior interventions for children with ASD benefit from increased intensity rather than duration; whereas, language interventions benefited from increased duration (Virues-Ortega, 2010). In coaching interventions used in EI, providers may benefit from setting short term goals that are achievable within 9 weeks to increase child adaptive behavior and participation. For example, eating or feeding, goals that involve decreasing food selectivity, and/or toileting goals that involve multiple components (bladder control, dressing) may require intensive focus from parents and 9 weeks is appropriate, then, to address one specific skill domain.

Another novel finding from the current study suggests that there were no significant differences found among families that had received in-home services previously to telehealth and those that had not. These findings coincide with other studies reporting high satisfaction with the use of telehealth by families and therapists (Wallisch et al., 2019; Zylstra, 2013); however, focus group results from families that qualified for EI prior to COVID-19 reported that caregivers preferred in-person visits (Yang et al., 2020). Current data shows that the pandemic likely shifted caregiver perceptions and readiness for engaging in telehealth, regardless of a family's previous participation in in-person services.

Lastly, data showed that some goals overlapped among disciplines (e.g., motor, feeding). In EI, providers use a team-based approach and collaboration. Multiple providers may work toward a similar family goal through the lens of their individual discipline. For example, if a family's goal relates to mealtime participation, an occupational therapy practitioner may focus on collaborating with the caregiver on increasing children's self-feeding skills whereas the speech-language pathologist may focus on increasing oral-motor skills. The commonalities demonstrated among goals by EI provider serve as evidence of the collaborative approach using their discipline-specific expertise to achieving family identified goals; the coaching approach likely increases parent capacity to reach goals.

## LIMITATIONS AND FUTURE DIRECTIONS

First, limitations of the current study include a lack of fidelity measure to track EI providers’ use of the coaching model. Second, each EI provider also had varying knowledge related to their professional training which was integrated within their telehealth sessions; therefore, intervention content varied by provider and by session. Third, we cannot account for any additional services the families were receiving during the intervention window. Fourth, the measures included in this study relied on parent report measures. Lastly, we did not include potential moderating factors in the statistical models such as child age, diagnosis, and potential influence of primary and secondary effects of COVID-19 on family functioning and well-being.

Future directions for research would include a larger sample size in order to assess outcomes among a large variety of families. This also includes the examination of factors related to state-wide EI adoption, sustainability, and large-scale measurement of how the coaching model is used when services are provided through telehealth. In order to facilitate large scale adoption of coaching via telehealth in practice, EI networks must consider factors associated with access to technology to ensure widespread access for all families needing services. Further, future research should use a systematic approach from an implementation science framework to better understand the uptake and adoption of evidence-based coaching intervention via telehealth in state-wide EI networks.
